# ﻿Large carrion and burying beetles evolved from Staphylinidae (Coleoptera, Staphylinidae, Silphinae): a review of the evidence

**DOI:** 10.3897/zookeys.1200.122835

**Published:** 2024-05-08

**Authors:** Derek S. Sikes, Margaret K. Thayer, Alfred F. Newton

**Affiliations:** 1 University of Alaska Museum / Department of Biology and Wildlife, University of Alaska Fairbanks, 1962 Yukon Dr., Fairbanks, Alaska, USA University of Alaska Fairbanks Fairbanks United States of America; 2 Negaunee Integrative Research Center, Field Museum of Natural History, 1400 South DuSable Lake Shore Drive, Chicago, Illinois, USA Negaunee Integrative Research Center, Field Museum of Natural History Chicago United States of America

**Keywords:** Monophyly, Nicrophorini, Nicrophorus, paraphyly, rove beetles, Silphidae, Silphini

## Abstract

Large carrion beetles (Silphidae) are the focus of ongoing behavioral ecology, forensic, ecological, conservation, evolutionary, systematic, and other research, and were recently reclassified as a subfamily of Staphylinidae. Twenty-three analyses in 21 publications spanning the years 1927–2023 that are relevant to the question of the evolutionary origin and taxonomic classification of Silphidae are reviewed. Most of these analyses (20) found Silphidae nested inside Staphylinidae (an average of 4.38 branches deep), two found Silphidae in an ambiguous position, and one found Silphidae outside Staphylinidae, as sister to Hydrophilidae. There is strong evidence supporting the hypothesis that large carrion beetles evolved from within Staphylinidae and good justification for their classification as the subfamily Silphinae of the megadiverse, and apparently now monophyletic, Staphylinidae. Considerable uncertainty remains regarding the interrelationships and monophyly of many staphylinid subfamilies. Nonetheless, the subfamily Tachyporinae was found to be the sister of Silphinae in more analyses (7) than any other subfamily.

“*Silphidae may instead be a sister group to Staphylinidae, or an isolated basal lineage within it, and its exact relationship to Staphylinidae* sensu latissimo *is in our opinion the most difficult remaining issue concerning the monophyly of Staphylinidae*.” – Grebennikov and Newton 2009

## ﻿Introduction

Paraphyly is a common classification error often resulting from a lineage evolving into a new ecological space that differs significantly from its closest relatives. Classic examples include tetrapods from fish (Irisarri and Meyer 2016), birds from dinosaurs (Feduccia 2002), hexapods from Crustacea (von Reumont et al. 2012), termites from Blattodea (Inward et al. 2007), and parasitic lice from Psocodea (Johnson et al. 2018). At the family level, and within beetles, a well-known and relevant example of paraphyly is that of the economically important bark beetles, formerly the family Scolytidae, now a subfamily within Curculionidae (Crowson 1955; Jordal et al. 2014). Often, the morphology of the group evolving into the new ecological space is so modified from its ancestral condition that the value of morphology for understanding the phylogenetic placement of the taxon is diminished. This led many early taxonomists to separate these groups into their own higher taxa with their sister lineages often enigmatically unknown. With advances in dataset types (e.g., larval morphology, molecules) and dataset sizes (e.g., phylogenomics), and phylogenetic methods, for the examples listed above and many more, researchers found taxonomic solutions to restore monophyly of the “parent” taxa by sinking the aberrant lineages into their parents. This yields a more phylogenetically accurate classification corresponding to the tree of life and employs best taxonomic practices (Vences et al. 2013) but can confuse people who have trouble envisioning these often-radical evolutionary transformations. To this list we propose to add the large carrion beetles having evolved from within Staphylinidae.

Large carrion beetles are a relatively well-known small monophyletic group of approximately 189 extant species worldwide. They have traditionally been treated as a family (Silphidae) with two subfamilies: Nicrophorinae and Silphinae (Sikes 2016). Their large body size (7–45 mm, usually 12–20 mm), low species richness, parental care (in the genus *Nicrophorus*), diverse ecology (primarily necrophagy), and ease of capture, identification, and culturing have made them a popular group for study in many fields of biology, e.g., behavior (Trumbo et al. 2016), ecology (Anderson 1982), conservation biology (Holloway and Schnell 1997), forensic entomology (Jakubec et al. 2019), and evolution (Ikeda et al. 2012). The group is well-known among the public and is one of the families most students who have taken a general entomology class have learned. The group is also taxonomically well-known (Anderson and Peck 1985; Peck and Anderson 1985; Sikes et al. 2002) with few new species expected, and each subfamily has received phylogenetic investigation (Dobler and Müller 2000; Ikeda et al. 2012; Sikes and Venables 2013). The family Staphylinidae, commonly known as rove beetles, is the largest family of life on earth, with 66,928 species grouped into one extinct and 34 extant (including Silphinae) subfamilies (Newton 2022). Most rove beetles are much smaller-bodied (< 1–35 mm, usually 2–8 mm) than large carrion beetles and primarily predatory, but also mycophagous and detritivorous with various minor exceptions (e.g., species of the genus *Aleochara* are parasitoids and *Eusphalerum* species, *inter alia*, feed on pollen).

Although there has long been consensus that Silphidae are monophyletic (Sikes 2016) and belong to the superfamily Staphylinoidea (e.g., Peck 2000), consensus was lacking regarding the relationship of Silphidae to Staphylinidae. An increasing number of phylogenetic analyses, using both morphological and molecular data, have found the lineage rooting inside Staphylinidae. Hatch (1927) was the first to formally classify the large carrion beetles as a subfamily of the Staphylinidae, but his proposed reclassification was not accepted by the scientific community. Almost a century later, Cai et al. (2022), based on their reanalysis of data from Zhang et al. (2018) and other studies that had found similar results, again formally classified the large carrion beetles as a subfamily of Staphylinidae. During the 95 years between these works, many phylogenetic studies on beetles have been published, some of which are relevant to the relationship of Silphidae to Staphylinidae. Herein we review chronologically, based on year of publication, works from 1927–2023 that are relevant to the question of how Silphidae are related to Staphylinidae. We aim to provide a concise summary of the evidence supporting the classification of large carrion beetles as a subfamily of Staphylinidae.

## ﻿Methods

We limit our review to works that were conducted in such a way that Silphidae could root inside or outside the Staphylinidae. Thus, we excluded works that used Silphidae as an outgroup of Staphylinidae (Zhang and Zhou 2013; Liu et al. 2021), used family-only terminals (Beutel and Leschen 2005), or included no non-staphylinids (Yamamoto 2021). We also excluded works that did not bring new data to bear on the question. Thus, we excluded works that were entirely re-analyses of datasets from prior works we review (Toussaint et al. 2016; Gusarov 2018; Cai et al. 2022) and all publications that simply treated Silphidae as a family without evolutionary analysis or other justification, following the consensus classification of the time. We also excluded works investigating Coleoptera or Staphyliniform evolution prior to the 1990s, such as Jeannel and Jarrige (1949), Coiffait (1972), Crowson (1981), and Naomi (1985), except for two that had novel findings regarding Silphidae (Hatch 1927; Lawrence and Newton 1982). Our goal was to focus on works that represent independent tests, using modern phylogenetic methods, of the hypothesis that Silphidae evolved from within Staphylinidae. None of these works were focused only on this question, however. Herein, we use the staphylinid subfamily name Silphinae in the new sense of Cai et al. (2022), which corresponds to the former family Silphidae (~ 189 species), not the former subfamily Silphinae (~ 119 species). We use Silphidae or Silphinae depending on context, but in all cases are referring to the same ~ 189 species. We use the current classification of Staphylinidae in our reference to the separate subfamilies Tachyporinae and Mycetoporinae (Yamamoto 2021).

For each study we provide information to help judge the robustness of the analysis and its findings relevant to the placement of Silphinae. We include the number and type of datasets used. Number of datasets was approximately equivalent to number of genes analyzed (we counted mitogenome analysis as 16 datasets because there are 15 non-tRNA genes in beetle mitochondrial genomes and we count all the tRNAs as a single gene because of their small size). Generally, phylogenetic accuracy increases with the number of genes and number of taxa used in an analysis. We indicate what type of analysis was performed (viz. non-algorithmic, parsimony, Maximum Likelihood, Bayesian). Using subfamilies as terminals, we provide a simplified figure, built using MESQUITE v. 3.6 (Maddison and Maddison 2018), depicting each work’s preferred phylogeny, limited to only Staphylinidae, for all but Korte et al. (2004). Korte et al. (2004) was the only study to find Silphinae external to Staphylinidae, so to depict their phylogeny we had to include non-staphylinid terminals. We provide a count of how many branches deep Silphinae join within Staphylinidae, based on our simplified tree figures. This count is equal to the number of branches that would need to collapse for Silphinae to be in a polytomy with basal Staphylinidae. We also provide a count of how many of these branches are strongly supported, using the branch support criteria of each analysis (if applicable). Based on Erixon et al. (2003) and Hoang et al. (2017) we interpreted strong branch support as Bayesian Posterior Probabilities ≥ 0.90, bootstrap values ≥ 84%, and ultrafast bootstrap values ≥ 95% and indicate well-supported branches on our figures. Finally, we provide counts of the total OTUs in each analysis, counts of the total Staphylinidae (including Silphinae) OTUs, and what percent of those OTUs were families of Staphylinoidea. Because there is no doubt that Silphidae evolved within the superfamily Staphylinoidea (Lawrence and Newton 1982), the strongest tests should have the diversity of this superfamily well represented in case Silphidae is more closely related to a non-staphylinid staphylinoid family. An analysis with many non-staphylinoid families represented but with the only staphylinoid families being Silphidae and Staphylinidae would be a weak test because these two families would be expected to join together regardless of the true relationship between them. Staphylinoidea currently contains six families besides Silphidae and Staphylinidae (Cai et al. 2022; Newton 2022), so we indicate which and provide a percentage of these six families (Agyrtidae, Colonidae, Hydraenidae, Jacobsoniidae, Leiodidae, Ptiliidae) represented in each analysis. Most works had a single dataset and corresponding preferred analysis, but some had multiple datasets and corresponding analyses, so we review 23 analyses from 21 works. If the authors did not indicate a preferred analysis and used multiple methods (parsimony, Bayesian, Maximum Likelihood) we chose to present and discuss their most statistically justified analysis (Bayesian or Maximum Likelihood). We also provide brief commentary on the findings of each study. Our primary goal is to provide a concise summary of the evidence for why the family Silphidae has been sunk into the family Staphylinidae, not to provide an in-depth review of each study’s strengths and weaknesses. Finally, because Hatch (1927) was the first to propose that Silphinae evolved from within Staphylinidae, for conciseness we sometimes refer to this as “Hatch’s (1927) hypothesis.”

## ﻿Results

**Figure 1.**
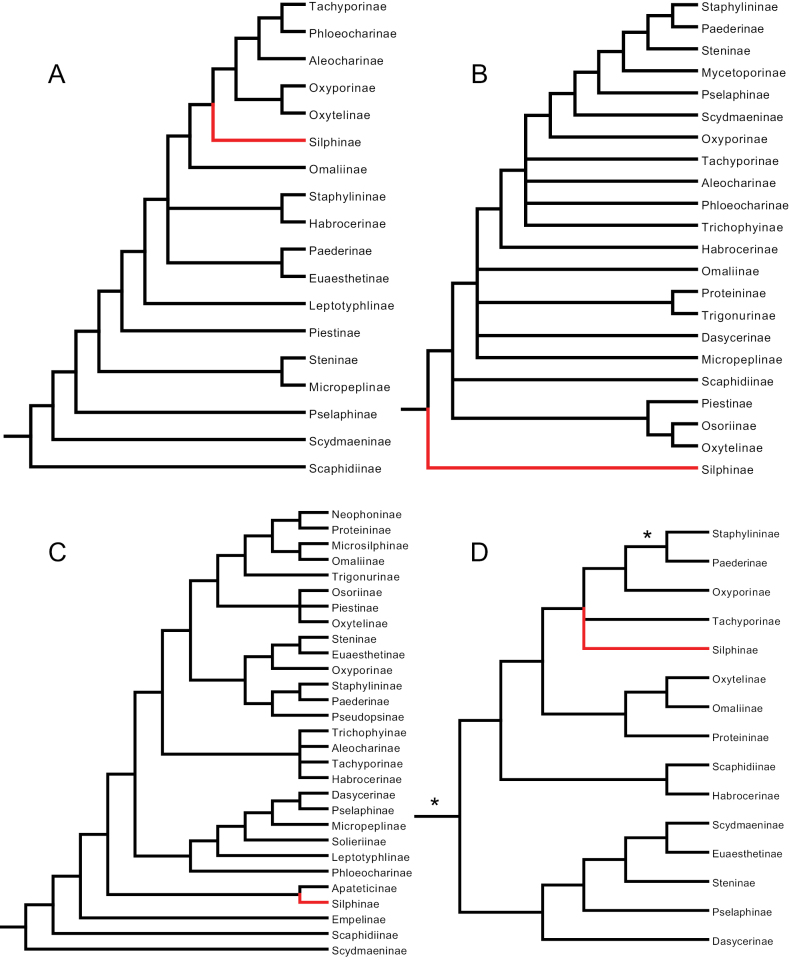
Simplified Staphylinidae phylogenies from **A**Hatch (1927) with non-staphylinids removed **B**Beutel and Molenda (1997: fig. 50), parsimony tree **C**Hansen (1997: fig. 5), parsimony tree, and **D**Ballard et al. (1998: fig. 3a), parsimony tree. Silphinae indicated in red, asterisks indicate well-supported branches.

Hatch (1927), using evolutionary taxonomic methods (non-algorithmic) and morphological characters, explicitly classified silphids as a subfamily of Staphylinidae nine “branches” deep. He was using a now antiquated concept of Silphidae that included current Silphinae and beetles since moved to their own families (Agyrtidae and Leiodidae). We believe he was the first to formally propose that silphids should be sunk into staphylinids and thus his work is historically significant for this review. A large section of his text and corresponding key was devoted to a discussion of characters supporting this change. His key was not artificial, that is, it was intended to reflect phylogeny by arranging taxa in a natural sequence with supporting “derivative characters” (akin to synapomorphies) indicated in his key. We have mapped the hierarchically nested taxa within his key to an interpretation of his intended phylogeny (Fig. 1A). He included 31 beetle OTUs, 19 of which were Staphylinidae, and included three additional staphylinoid families: Ptiliidae, Leiodidae, and Colonidae (Table 1).

**Table 1.** Analyses relevant to the evolutionary origin of the Silphinae. In/out: whether Silphinae joined inside Staphylinidae. Depth: number of branches that would need to collapse for Silphinae to fall into a polytomy with basal Staphylinidae. Depth Strength: number of such branches well supported. Methods: NA, non-algorithmic; MP, maximum parsimony; ML, Maximum Likelihood; BI, Bayesian Inference. % St-oidea: Percentage of the six families of Staphylinoidea represented, not counting Silphidae and Staphylinidae.

	Analysis	Year	In/out	Datasets/ genes	Data description	Method(s)	Depth	Depth Strength	OTUs	Staph OTUs	% St-oidea	Sister to Silphinae
1	Hatch	1927	in	1	morphology	NA	+9	n/a	31	19	50	5 subfam. incl. Tachyporinae
2	Lawrence and Newton	1982	in	1	morphology	NA	n/a	n/a	n/a	n/a	100	n/a
3	Beutel and Molenda	1997	?	1	morphology	MP	1	n/a	29	22	50	All remaining Staphylinidae
4	Hansen	1997	in	1	morphology	MP	+5	n/a	37	22	83	Apateticinae
5	Ballard et al.	1998	in	3	rDNA (12S), mtDNA (Cyt b), morphology	MP	+4	+1	25	23	33	In polytomy with Tachyporinae
6	Korte et al.	2004	out	2	rDNA (18S, 28S)	MP, BI	n/a	n/a	35	6	33	n/a
7	Caterino et al.	2005	in	2	rDNA (18S) and morphology	MP, ML, BI	+3	+1	105	35	67	Phloeocharinae
8	Hunt et al.	2007	in	3	rDNA (18S), mt-rDNA (16S) and COI	MP, BI	+6	0	340	20	67	Tachyporinae
9	Grebennikov and Newton	2009	in	1	rDNA (18S)	MP, NJ, BI	+5	+2	93	75	67	Tachyporinae
10	Lawrence et al.	2011	in	1	morphology	MP	+4	0	359	11	100	Tachyporinae+Staphylininae
11	Grebennikov and Newton	2012	?	1	morphology	MP	1	0	36	34	33	All remaining Staphylinidae
12	Bocak et al.	2014	in	4	rDNA (18S, 28S), mtDNA (rrnL, COI)	ML	+8	0?	8,441	349	67	Tachyporinae
13	McKenna et al.	2015	in	2	rDNA (28S), CAD	BI, ML	+3	+3	282	51	83	Tachyporinae
14	Timmermans et al.	2016	in	16	mitogenomes	ML, BI	+4	+3	245	11	33	Habrocerinae and Aleocharinae (in part)
15	Zhang et al.	2018	in	95	protDNA (Amino Acids)	ML, BI	+5	+2	373	16	83	Apateticinae, Scaphidiinae, and Osoriinae
16	Kypke (PhD diss: fig4)	2018	in	993	genomics	ML	+3	+3	33	25	50	Oxytelinae
17	Kypke (PhD diss: fig. 5)	2018	in	1,033	genomics	ML	+2	0	57	41	83	many subfamilies
18	McKenna et al.	2019	in	4,818	genomics	ML	+2	+2	146	4	50	Staphylininae
19	McKenna et al.	2019	in	89	DNA	ML	+4	+2	521	20	83	Apateticinae, Scaphidiinae, and Osoriinae
20	Lü et al.	2019	in	6	nDNA (CAD, Wg, 28S, 18S), mtDNA (Cyt b, 16S)	ML	+6	0	664	614	83	Tachyporinae
21	Cai and Li	2021	in	13	mtDNA (protein coding genes only)	ML	+4	0	40	11	50	Staphylininae
22	Song et al.	2021	in	16	mitogenomes	BI	+6	+3	107	95	17	Tachyporinae (in part)
23	Zhao et al.	2022	in	16	mitogenomes	ML	+7	+5	93	85	50	Tachyporinae (in part)

Lawrence and Newton (1982), using cladistic reasoning (but non-algorithmic) and phenotypic characters (morphology and behavior) of adults and larvae, delimited groups of staphylinid subfamilies and commented on the family Silphidae. Their “staphylinine group” contains many mostly predatory species which share the behavior of extraoral digestion, among other characters. They added that the families Silphidae and Scydmaenidae share traits with this subfamily group and these families may have evolved from Staphylinidae. They did not include a phylogenetic analysis in their work, but their comprehensive review of staphylinid higher taxon relationships warrants review here. This work is historically important in being the first of the modern phylogenetic era (post-Hennig) to suggest that Silphidae may belong inside Staphylinidae. We categorized their findings as support for Hatch’s (1927) hypothesis because they included potential synapomorphies shared by Silphinae and Staphylinidae (Table 1). They considered all beetle families known at the time, including all current staphylinoid families, but did not mention Jacobsoniidae in the context of Staphylinoidea because it had yet to be recognized as a member of Staphylinoidea.

Beutel and Molenda (1997) investigated staphylinoid relationships using internal and external larval head morphology and parsimony methods. They, or Hansen (1997), were the first to use a modern algorithmic phylogenetic method that addressed this question. Silphidae was inferred to be the sister lineage to the remaining Staphylinidae (Fig. 1B). We categorized this finding as ambiguous because it could also be interpreted as the Silphinae being the basal lineage within Staphylinidae, depending on one’s delimitation of Staphylinidae. This work therefore does not reject Hatch’s (1927) hypothesis. They included 29 OTUs, 22 of which were Staphylinidae, and included three additional staphylinoid families: Hydraenidae, Leiodidae, and Agyrtidae (Table 1).

Note that the larva identified as “*Euaesthetus* sp.” in Beutel and Molenda (1997) was misidentified and actually is Mycetoporinae, as can be seen by comparing their figures to those of larvae of both these groups in Kasule (1966). These groups are superficially similar in having six stemmata arranged in a circle on each side of the head and lacking a very distinct labrum but differ in several characters (listed here with reference to the figures in Kasule 1966). *Euaesthetus* larvae have a distinct antebasal neck constriction (fig. 58), the labrum completely fused to the head capsule to form a nasale bearing one or more pairs of apical teeth (fig. 57), and a maxilla with an extremely small mala that extends barely as far as the first palpomere (fig. 60). In contrast, mycetoporines lack an antebasal neck constriction (fig. 38) and have an indistinctly articulated labrum without anterior teeth (fig. 38) and a maxilla with a very large mala that extends to about the middle of the third palpomere (fig. 40). Beutel and Molenda (1997) also miscoded the labrum of this larva as completely fused (their character state 7-2) rather than partly fused (state 7-1) to the head, which probably affected the placement of the larva in their tree. In our representation of their tree here (Fig. 1B) we therefore replaced the name Euaesthetinae with Mycetoporinae.

Hansen (1997) conducted a phylogenetic analysis of staphyliniform beetles using morphology of adults and immatures with parsimony methods. Hansen’s preferred tree has Silphinae five branches deep and sister to Apateticinae, within what modern workers would define as Staphylinidae, with Empelinae, Scaphidiinae, and Scydmaeninae joining closer to the base than Silphinae (Fig. 1C). Although Hansen (1997) did not use a statistical method such as bootstrapping to assess branch support, he did indicate the number of character state changes (apomorphies) estimated for each branch. His root branch of Staphylinidae had 17 apomorphies inferred. About this clade (Staphylinidae in the modern sense) he wrote “A very well defined and undoubtedly monophyletic group, characterized by several very weighty apomorphies, some of which are unique.” He included 37 OTUs, 22 of which were Staphylinidae, and included five additional staphylinoid families: Ptiliidae, Hydraenidae, Leiodidae, Colonidae, and Agyrtidae (Table 1).

Ballard et al. (1998) used three datasets, one of adult morphology and two molecular (12S ribosomal RNA and cytochrome b mitochondrial DNA) using parsimony methods to infer relationships among 25 staphylinoid OTUs, 23 of which were Staphylinidae, and included two additional staphylinoid families: Leiodidae and Agyrtidae. They were the first to bring molecular data to bear on this question. Their preferred tree has Silphinae four branches deep within Staphylinidae in a polytomy with Tachyporinae among other subfamilies (Fig. 1D). One of these four branches was well supported. The only silphine they included, *Oiceoptoma*, was sister to the genus *Tachinus* (Tachyporinae) and supported by a 70% bootstrap in their conditional data combination bootstrap consensus tree (not shown).

**Figure 2.**
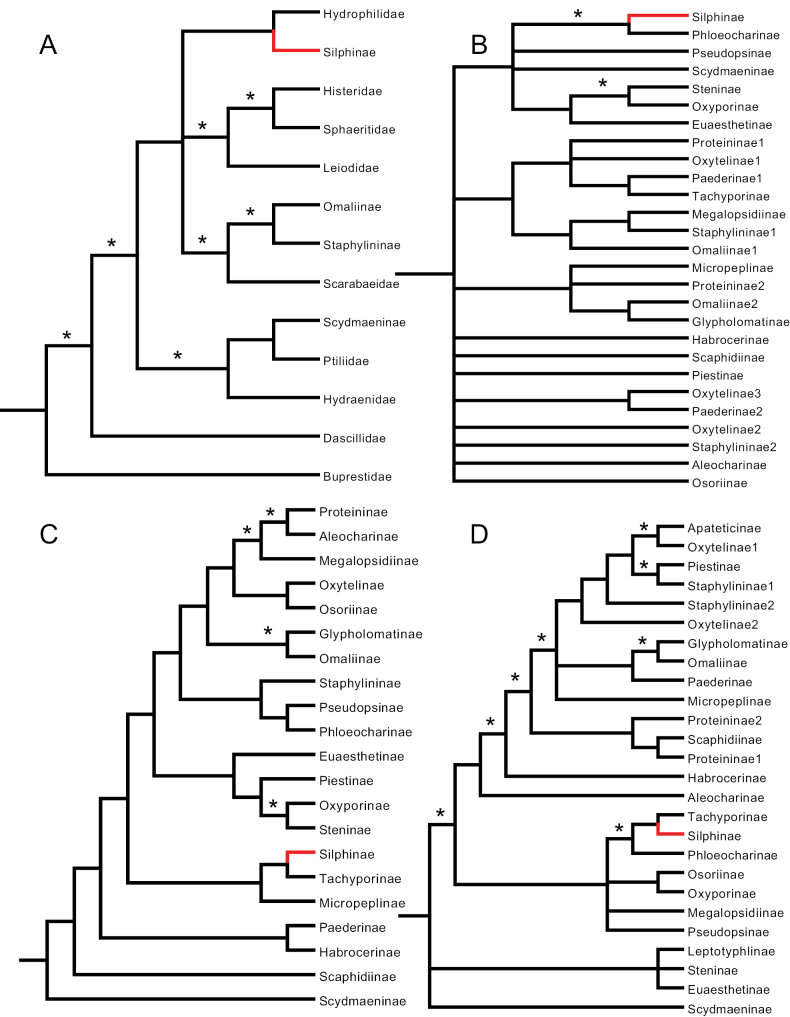
Simplified Staphylinidae phylogenies from **A**Korte et al. (2004: fig. 3), Bayesian tree **B**Caterino et al. (2005: fig. 5) Bayesian tree **C**Hunt et al. (2007: fig. 2) Bayesian tree, with non-staphylinids removed, and **D**Grebennikov and Newton (2009: fig. 12) Bayesian tree, with non-staphylinids removed. Because Korte et al. (2004) was the only study to find the Silphinae external to the Staphylinidae**A** is our only figure that includes non-staphylinid OTUs. Silphinae indicated in red, asterisks indicate well-supported branches.

Korte et al. (2004) used two molecular datasets of nuclear ribosomal DNA (18S, 28S) to infer the relationships among 35 beetle OTUs, 6 of which were Staphylinidae. They included two additional staphylinoid families: Hydraenidae and Leiodidae, using parsimony and Bayesian methods (Table 1). Their Bayesian analysis found a polyphyletic Staphylinidae with both Silphinae (monophyletic) and Scydmaeninae joined as sister taxa to non-staphylinid lineages, viz. Hydrophilidae and Ptiliidae, respectively (Fig. 2A). Of all the works we review herein, this is the only one that found Silphinae neither inside nor as the sister group of Staphylinidae. Ribosomal DNA is notoriously hard to align properly, especially when secondary structure is not used (Buckley et al. 2000). Although these authors did due diligence in their use of a variety of phylogenetic methods that were well-justified at the time, and even explored a variety of alignments (but did not use secondary structure), some of their results were not entirely credible. They stated as much in the final sentence of their abstract “Some results, such as a placement of Silphidae as subordinate group of Hydraenidae (parsimony tree), or a sistergroup relationship between Ptiliidae and Scydmaenidae, appear unlikely from a morphological point of view.”

Caterino et al. (2005) used two datasets, one of morphological characters derived and slightly modified from Hansen (1997) and the other of nuclear ribosomal DNA (18S) to infer relationships among 105 beetle OTUs, 35 of which were Staphylinidae. They included four additional staphylinoid families: Ptiliidae, Hydraenidae, Leiodidae, and Agyrtidae, using parsimony, Maximum Likelihood, and Bayesian methods (Table 1). Their Bayesian analysis recovered a monophyletic Staphylinidae with a monophyletic Silphinae three branches deep, one of which was well supported (Fig. 2B), with Phloeocharinae sister to Silphinae.

Hunt et al. (2007) used three molecular datasets, one of nuclear ribosomal DNA (18S), one of mitochondrial ribosomal DNA (16S), and one of protein-coding mitochondrial DNA (COI) to infer relationships using parsimony and Bayesian methods among 340 beetle OTUs, 20 of which were Staphylinidae, and four additional staphylinoid families: Ptiliidae, Hydraenidae, Leiodidae, and Agyrtidae, (Table 1). Their Bayesian analysis recovered a polyphyletic Staphylinidae with Silphinae six branches deep; none of these branches were well supported (Fig. 2C). This analysis found Tachyporinae sister to Silphinae.

Grebennikov and Newton (2009) used one molecular dataset of nuclear ribosomal DNA (18S) to infer relationships among 93 beetle OTUs, 75 of which were Staphylinidae, and included four additional staphylinoid families: Ptiliidae, Hydraenidae, Leiodidae, and Agyrtidae, using parsimony, neighbor-joining, and Bayesian methods (Table 1). They also prepared and analyzed a morphological dataset, but they did not do a combined molecular-morphological analysis. Because their morphological data were improved upon and formed the basis for their later work (Grebennikov and Newton 2012) we discuss their morphological findings later. Their 18S Bayesian analysis found a monophyletic Silphinae five branches deep, two of which were well supported (Fig. 2D). This was within a Staphylinidae made paraphyletic by Ptiliidae (not shown). They, like Hunt et al. (2007), found Tachyporinae sister to Silphinae.

**Figure 3.**
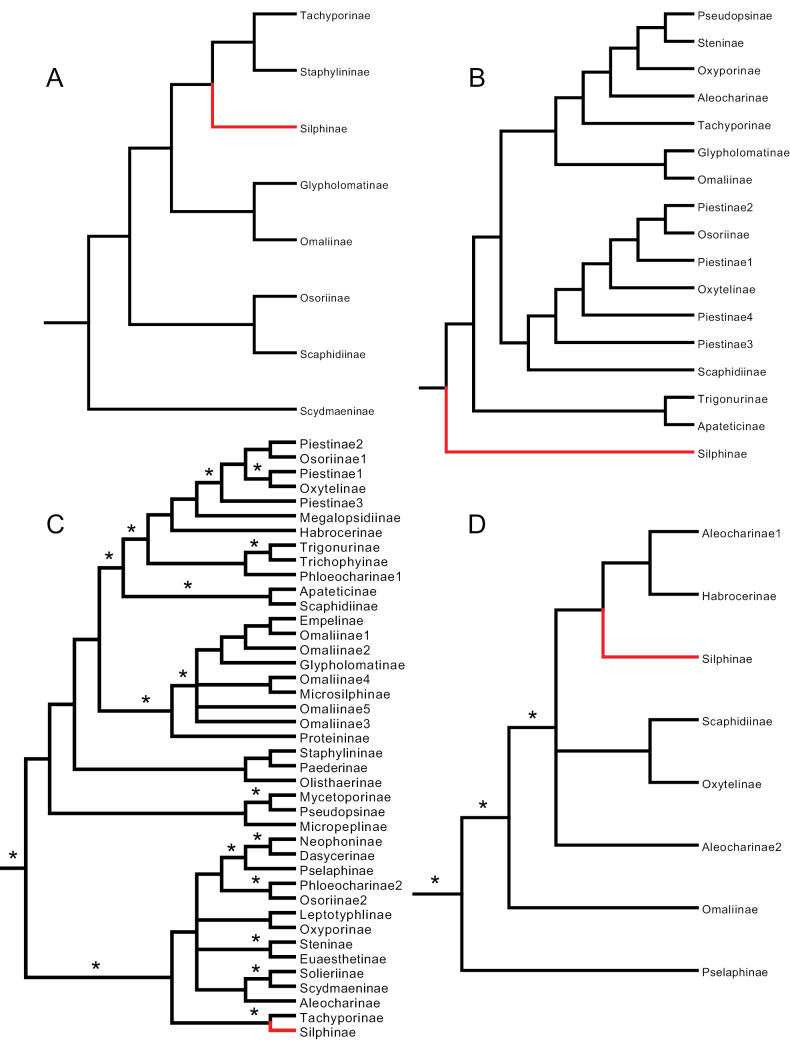
Simplified Staphylinidae phylogenies from **A**Lawrence et al. (2011: cladogram 2), parsimony tree **B**Grebennikov and Newton (2012: fig. 3), parsimony tree **C**McKenna et al. (2015: fig. 3), Bayesian tree, **D**Timmermans et al. (2016: fig. 1), Bayesian tree. Silphinae indicated in red, asterisks indicate well-supported branches.

Lawrence et al. (2011) used a single morphological dataset and parsimony methods to infer relationships among 359 beetle OTUs, 11 of which were Staphylinidae, and included all six additional staphylinoid families: Ptiliidae, Hydraenidae, Leiodidae, Colonidae, Agyrtidae, and Jacobsoniidae (Table 1). Their analysis recovered a monophyletic Silphinae four branches deep within a monophyletic Staphylinidae, as sister to a clade of Tachyporinae and Staphylininae (Fig. 3A). None of these branches were well supported. This and Hansen (1997) were the only morphology-only studies to find the Silphinae rooting deeply inside the Staphylinidae since Hatch (1927), rather than as a sister lineage.

Grebennikov and Newton (2012), who built upon the morphological dataset they prepared for their 2009 study, used this single dataset and parsimony methods to infer relationships among 36 beetle OTUs, 34 of which were Staphylinidae, and included two additional staphylinoid families: Leiodidae and Agyrtidae. Their analysis inferred Silphinae to be the sister lineage to the remaining Staphylinidae (Fig. 3B). We categorized this finding as ambiguous because it could also be interpreted as Silphinae being the basal lineage within Staphylinidae, depending on how the family Staphylinidae was delimited. This work therefore does not reject the hypothesis of Silphinae as Staphylinidae. The synapomorphies they inferred and nicely illustrated for the branch uniting Silphinae and Staphylinidae can be used to diagnose Staphylinidae in its current sense (see discussion). Although these authors used parsimony bootstrapping to assess branch support, they did not present bootstrap values on their preferred analysis tree (their fig. 3) and none of the bootstrap values presented in their table 3 for their preferred analysis (#9) were above 83% for any of the non-terminal branches we depict in Fig. 3B.

Bocak et al. (2014) used four datasets, two nuclear (ribosomal 18S, 28S) and two mitochondrial (ribosomal rrnL and protein-coding COI) to infer relationships using Maximum Likelihood methods among 8,441 beetle OTUs, 349 of which were Staphylinidae. They included four additional staphylinoid families: Ptiliidae, Hydraenidae, Leiodidae, and Agyrtidae (Table 1). We were not able to determine if they included Colonidae. Their analysis found a monophyletic Silphinae nested eight branches deep in Staphylinidae and sister to Tachyporinae. However, we were unable to determine the branch support of these branches, or if these authors calculated branch support. We were unable to reconstruct a simplified phylogeny from this work because of the unintelligible coding system they used to label branch tips and the lack of a taxa-included list. This work represents the largest beetle taxon sampling of those we have reviewed.

McKenna et al. (2015) used two datasets, nuclear ribosomal DNA (28S), and nuclear protein-coding DNA (CAD), to infer relationships using Maximum Likelihood and Bayesian methods among 282 beetle OTUs, 51 of which were Staphylinidae, and included five additional staphylinoid families: Ptiliidae, Hydraenidae, Leiodidae, Colonidae, and Agyrtidae (Table 1). They found a monophyletic Silphinae nested three branches deep in a monophyletic Staphylinidae as sister to Tachyporinae (Fig. 3C). All three branches were strongly supported in their Bayesian analysis but weakly supported in their Maximum Likelihood analysis.

Timmermans et al. (2016) used entire mitochondrial genomes, which contain 16 genes (13 protein coding and 2 ribosomal), and 22 transfer RNAs (which we treat as one gene-equivalent ‘dataset’ because of the small size of tRNAs) to infer the relationships using Maximum Likelihood and Bayesian methods of 245 beetle OTUs, 11 of which were Staphylinidae; they included two additional staphylinoid families: Leiodidae and Agyrtidae (Table 1). Their results had their one Silphinae (*Necrophila*) nested four branches deep in a monophyletic Staphylinidae as sister to a clade of Habrocerinae and some Aleocharinae (Fig. 3D). Three of these branches were well supported. Mitochondrial DNA in animals evolves faster than nuclear DNA, so is easier to use to infer recent splits (Avise 1986). Ancient splits are more challenging to resolve properly in mitochondrial DNA-only analyses and require careful model specification (Cameron 2014), as Timmermans et al. (2016) appear to have done.

**Figure 4.**
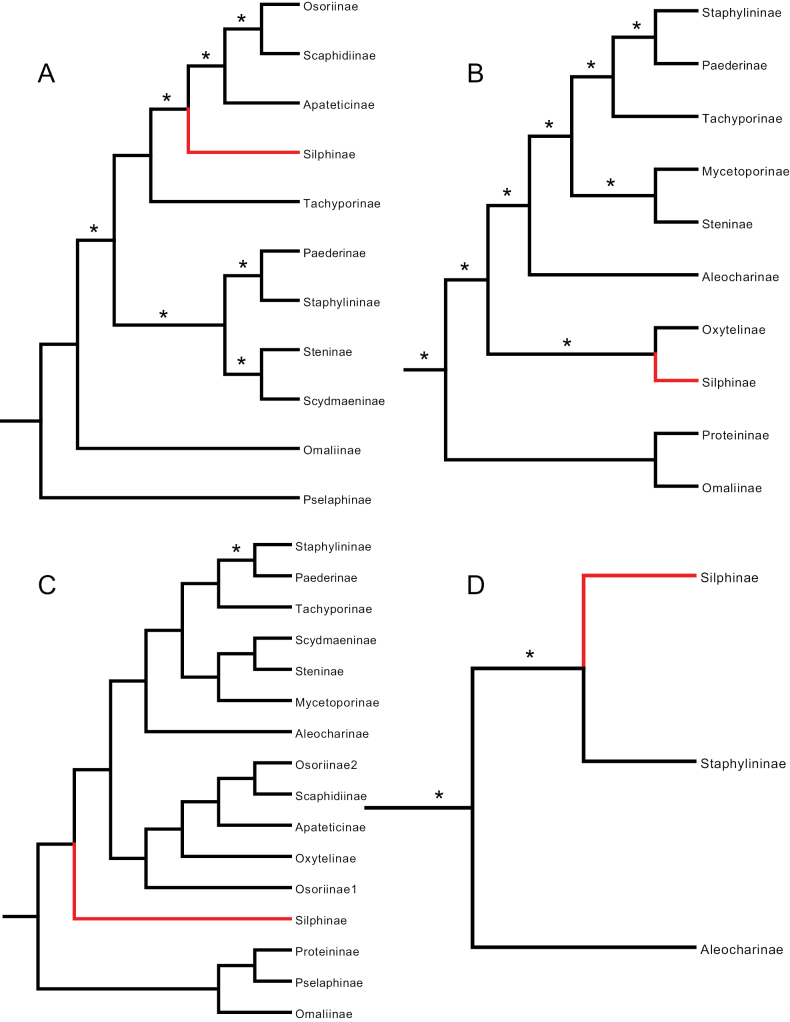
Simplified Staphylinidae phylogenies from **A**Zhang et al. (2018: fig. 2), ML+Bayesian tree **B**Kypke (2018: fig. 4), ML tree **C**Kypke (2018: fig. 5), ML tree **D**McKenna et al. (2019: fig. 1), ML tree. Silphinae indicated in red, asterisks indicate well-supported branches.

Zhang et al. (2018) also used phylogenomic methods. They built a dataset of 95 protein coding nuclear genes and analyzed their amino acid sequences using Maximum Likelihood and Bayesian methods to infer the relationships of 373 beetle OTUs, 16 of which were Staphylinidae. They included five additional staphylinoid families: Ptiliidae, Hydraenidae, Leiodidae, Agyrtidae, and Jacobsoniidae (Table 1). Their analysis found a monophyletic Silphinae nested five branches deep within a monophyletic Staphylinidae, as sister to a clade containing Apateticinae, Scaphidiinae, and Osoriinae (Fig. 4A). Two of these five branches were well supported. Tachyporinae was the sister group to the Silphinae et al. clade, though with weak support.

Kypke (2018) conducted two analyses relevant to Hatch’s (1927) hypothesis for her dissertation. She was one of the first to apply a phylogenomics approach to inference of staphylinoid relationships. One of her analyses used 993 genes and Maximum Likelihood methods to infer the relationships of 33 OTUs, 25 of which were Staphylinidae. She included three additional staphylinoid families: Hydraenidae, Leiodidae, and Agyrtidae (Table 1). This phylogeny had a monophyletic Silphinae three branches deep, sister to Oxytelinae within a monophyletic Staphylinidae (Fig. 4B). All three of these branches were well supported. Her second analysis used 1,033 genes to infer the relationships of 57 OTUs, 41 of which were Staphylinidae. In this analysis she included five additional staphylinoid families: Ptiliidae, Hydraenidae, Leiodidae, Agyrtidae, and Jacobsoniidae (Table 1). This phylogeny found a monophyletic Silphinae two branches deep but as sister to a large clade of many subfamilies (Fig. 4C). Neither of these two branches was well supported.

**Figure 5.**
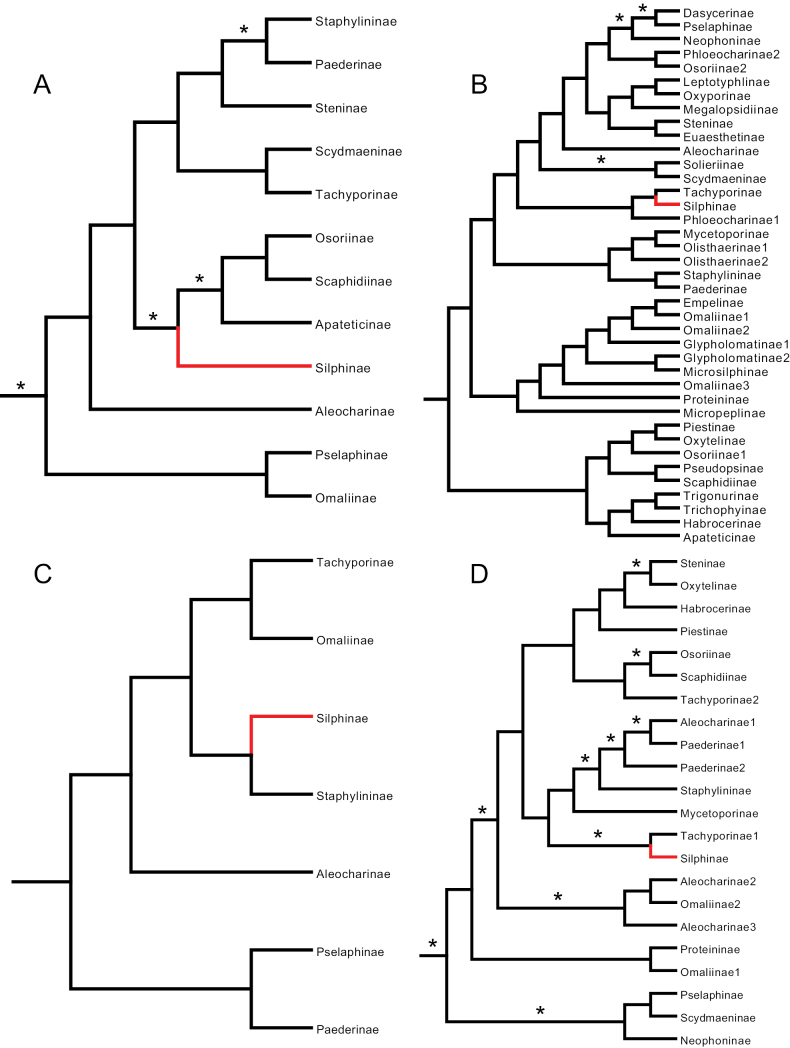
Simplified Staphylinidae phylogenies from **A**McKenna et al. (2019: fig. S10), ML tree **B**Lü et al. (2019: fig. S4), ML tree **C**Cai and Li (2021: fig. 1), ML tree **D**Song et al. (2021: fig. 1), Bayesian tree. Silphinae indicated in red, asterisks indicate well-supported branches.

McKenna et al. (2019) performed two phylogenomic analyses relevant to the evolutionary origin of the Silphinae. Their first had the largest dataset size of all the studies we review, 4,818 genes, but the smallest sampling of Staphylinidae (four OTUs representing three subfamilies) among their total taxon sample of 146 beetle OTUs. They included three additional staphylinoid families: Hydraenidae, Ptiliidae, and Jacobsoniidae. This analysis used Maximum Likelihood methods and found a monophyletic Silphinae nested two branches deep, as sister to Staphylininae, within a monophyletic Staphylinidae (Fig. 4D). Unsurprisingly, given their enormous dataset, both branches were well supported. Their second analysis, presented in the appendix of their paper, had better taxon sampling with 521 beetle OTUs, 20 of which were Staphylinidae, and included five additional staphylinoid families: Ptiliidae, Hydraenidae, Leiodidae, Agyrtidae, and Jacobsoniidae. They used 89 genes analyzed by Maximum Likelihood methods (Table 1). This analysis found a monophyletic Silphinae nested four branches deep within a monophyletic Staphylinidae, as sister to a clade containing Apateticinae, Scaphidiinae, and Osoriinae (Fig. 5A), similar to the finding of Zhang et al. (2018). Two of these four branches were well supported.

Lü et al. (2019) used six genes in total, four nuclear (CAD, Wg, 28S, 18S) and two mitochondrial (Cyt b, 16S) to infer relationships among 664 beetle OTUs, 614 of which were Staphylinidae, representing the largest staphylinid taxon sampling of any of our reviewed works (Table 1). They included five additional staphylinoid families: Ptiliidae, Hydraenidae, Leiodidae, Colonidae, and Agyrtidae. Using Maximum Likelihood methods, they found a monophyletic Silphinae nested six branches deep within a monophyletic Staphylinidae (Fig. 5B), as sister to Tachyporinae. However, none of these six branches were well supported.

Cai and Li (2021) used a dataset of all 13 protein-coding mitochondrial genes analyzed with Maximum Likelihood methods to infer the relationships of 40 beetle OTUs, 11 of which were Staphylinidae. They included three additional staphylinoid families: Ptiliidae, Hydraenidae, and Leiodidae (Table 1). Their analysis found a monophyletic Silphinae as sister to Staphylininae, nested four branches deep within Staphylinidae (Fig. 5C), but none of these branches were well supported.

Song et al. (2021), like Timmermans et al. (2016), used entire mitochondrial genomes analyzed using Bayesian methods to infer the relationships of 107 beetle OTUs, 95 of which were Staphylinidae, and included only one additional staphylinoid family, Leiodidae (Table 1). Their analysis, which had numerous genera assigned to the wrong subfamilies (corrected in our figure), found a monophyletic Silphinae as sister to part of Tachyporinae nested six branches deep in a monophyletic Staphylinidae (Fig. 5D). Three of these six branches were well supported.

**Figure 6.**
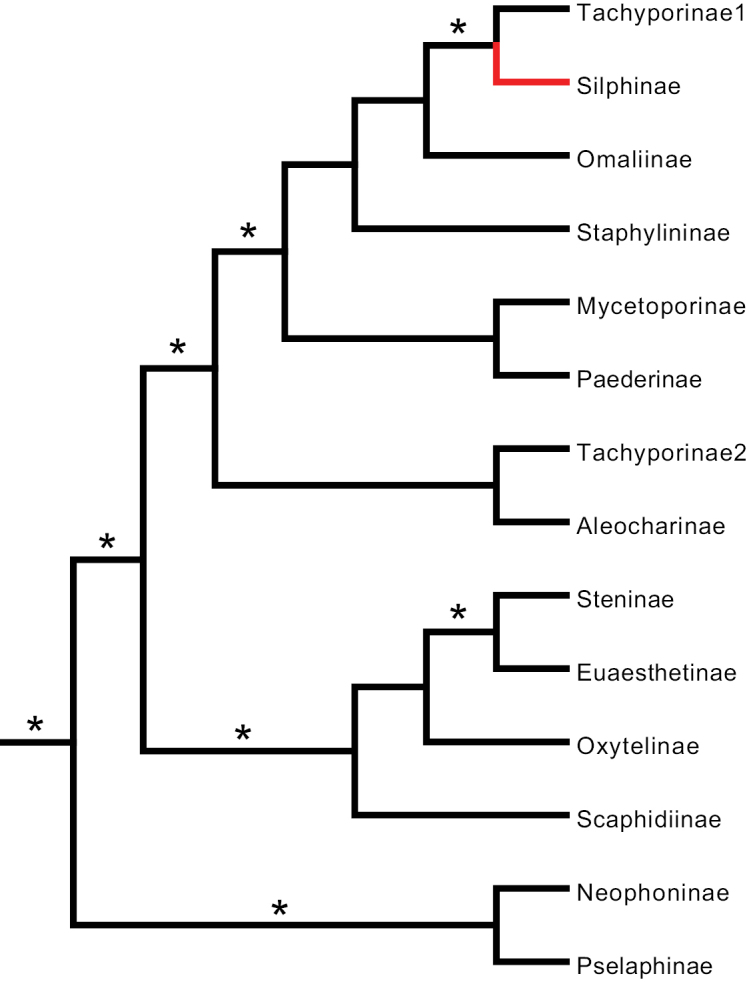
Simplified Staphylinidae phylogeny from Zhao et al. (2022: fig. 5a), ML tree. Silphinae indicated in red, asterisks indicate well-supported branches.

Zhao et al. (2022) used entire mitochondrial genomes and Maximum Likelihood methods to infer the relationships among 93 beetle OTUs, 85 of which were Staphylinidae. They included three additional staphylinoid families: Ptiliidae, Hydraenidae, and Leiodidae (Table 1). They conducted four similar analyses and did not select a preferred tree. We selected their best-supported tree (their fig. 5a) to illustrate and discuss. All four analyses found Silphinae inside Staphylinidae, and this one found a monophyletic Silphinae as sister to part of Tachyporinae, nested seven branches deep within Staphylinidae (Fig. 6). Five of these seven branches were well supported.

## ﻿Discussion

Considering all 23 analyses, it is apparent that the data type, number of genes, and analysis method do not matter: 97% failed to reject Hatch’s (1927) hypothesis. The only analysis to reject it was Korte et al. (2004), in which the authors themselves expressed reservations about the reliability of their results. Two analyses found ambiguous results, both based on morphological data (Beutel and Molenda 1997; Grebennikov and Newton 2012). These two found Silphinae as sister to Staphylinidae, which could be interpreted as indicating these taxa share a staphylinid ancestor and thus do not reject Hatch’s (1927) hypothesis. As mentioned in the introduction, when a lineage evolves into a new ecological space (in this case, necrophagy), the associated morphological changes can be so dramatic that phylogenetic inference with morphological data alone can be challenging because synapomorphies have evolved further into autapomorphies. It is thus not surprising that two of the four morphology-only analyses did not find Silphinae inside the Staphylinidae, as most of the molecular studies did. It is more surprising that the other two morphology-only studies (Hansen 1997; Lawrence et al. 2011) found Silphinae nested within the Staphylinidae.

With analyses ranging from a single gene to 4,818 genes and having a range of staphylinid OTUs from four to 614 with an average of 71.5, including a mega-analysis with 8,441 beetle OTUs, and most analyses including three or more of the six additional staphylinoid families, the evidence is strong for Hatch’s (1927) hypothesis. There were ample independent opportunities to reject it. However, there is still room to improve. Some of these analyses had enormous dataset sizes and others enormous taxon sampling, but none had both.

### ﻿Could all these independent analyses be wrong about the Silphinae rooting inside the Staphylinidae?

We do not think so. Systematic errors that are known to reduce phylogenetic accuracy, such as long branch attraction and other forms of model misspecification, do not seem to explain these results – particularly since some were based on morphological data and many used inference methods (e.g., Maximum Likelihood) known not to be predisposed to such biases. Could we have missed a significant number of publications that found contrary results? We do not think so. It is possible we have missed some analyses but doubt we have missed any large-scale, well-done, and relevant phylogenetic works that fit our criteria for inclusion and rejected Hatch’s (1927) hypothesis.

### ﻿Sister taxon to the Silphinae?

The reviewed analyses found a variety of different possible sister taxa of Silphinae, but Tachyporinae was found to be the sister group more often than any other subfamily (7 times in 19 analyses that included Tachyporinae). Three analyses did not include any Tachyporinae (Korte et al. 2004; Timmermans et al. 2016; McKenna et al. 2019: analysis 1) and Lawrence and Newton (1982) did not infer a tree. Although found as the sister group of Silphinae more often than any other (and three of these seven times were well supported branches), it is odd that, if Tachyporinae is the actual sister group, why this was not found in the large-dataset phylogenomic studies (e.g., Zhang et al. 2018; Kypke 2018).

Considerable uncertainty remains in our understanding of the intrafamilial relationships of the Staphylinidae. Although costly and difficult to accomplish, it would be ideal to have an analysis with hundreds of Staphylinidae representing all the subfamilies and most tribes, many outgroups including all staphylinoid families, hundreds of genes, as well as a morphological dataset, and no missing data. Given the apparent conflicting phylogenetic signal among many of the analyses we review, we suspect that even with such an ideal study design, the analysis will encounter many difficult challenges.

### ﻿Monophyletic Staphylinidae?

We conclude that with the addition of Silphinae as the 34^th^ subfamily of Staphylinidae, this megadiverse family is finally monophyletic. Grebennikov and Newton’s (2012) morphological investigation included 18 synapomorphies for Staphylinidae in this modern sense that separate this clade from closely related staphylinoids like agyrtids and leiodids (character #-state#: 8-0, 10-0, 13-1, 22-2, 38-0, 44-0, 45-1, 48-0, 66-0, 138-1, 160-2, 171-1, 218-0, 219-0, 228-0, 231-1, 247-1, 250-1). Seven of the most promising are: (44-0): Larval mandible lacking a molar lobe; (160-2): Adult with truncate elytra generally exposing 3+ terga; (171-1): Adult hind wing costal hinge present proximal to radial sector; (218-0, 219-0): Adult lacking wing-folding setal patches on terga VI–VII; (231-1): Adult abdominal intersegmental membranes with minute sclerites (though lost in some subfamilies); (250-1): Aedeagus with large basal bulb, small foramen. As discussed by Lawrence and Newton (1982) and Grebennikov and Newton (2012), many of these adult characters are probably functionally correlated with shortened elytra and the resultant exposure of multiple abdominal segments that need protection (hence the minute sclerites of the intersegmental membranes) and wings that need to be folded more compactly under the reduced elytra (hence the novel, more basal hinge). The aedeagal and larval characters, however, have no such apparent correlation with elytral length, and provide independent morphological confirmation that Staphylinidae including Silphinae is a monophyletic group, as suggested by the many molecular phylogenetic studies we review above.

### ﻿Fossil record

Fossils were not formally included in any of the phylogenetic analyses discussed above, even those based at least in part on morphology. This is partly because of the lack, until recently, of fossils that are adequately preserved and clearly attributable to Silphinae until the mid–late Tertiary (ca 35 Mya or younger), when fossils resembling or placed in modern genera of Silphinae appear (e.g., Cai et al. 2014, Chatzimanolis 2018). This situation has changed with the recent discovery of well-preserved compression fossils from the mid-Jurassic and early Cretaceous of China and South Korea, and mid-Cretaceous amber fossils from Myanmar, reviewed by Grebennikov and Newton (2012), Cai et al. (2014) and Sohn and Nam (2021). These discoveries encourage us to briefly discuss whether these fossils lend support to, or help refute, the general conclusion of our review of modern phylogenetic studies above that silphines are derived from within Staphylinidae. Only one of these Mesozoic silphine fossils, *Cretosajajinjuensis* Sohn and Nam from the early Jurassic of South Korea, is formally named (Sohn and Nam 2021), but the others are extensively described, illustrated, and discussed in the reviews of Cai et al. (2014) and Sohn and Nam (2021), on which the following comments are based. The earliest fossils, from the mid-Jurassic Daohugou Formation in China (ca. 165 Mya), closely resemble in habitus and many other characters small specimens of Nicrophorini, with strongly truncate elytra exposing at least four abdominal segments dorsally. They differ from modern Nicrophorini in being smaller (6.5–13.5 mm), with a more weakly developed antennal club (resembling modern Silphini), and notably lack any trace of stridulatory files on the abdominal terga. The early Cretaceous fossils, from the Yixian Formation of northeastern China and Jinju Formation of South Korea (both ca 125 Mya) closely resemble the Jurassic fossils in habitus and many structures, but have a more strongly developed antennal club resembling that of the modern nicrophorine genus *Ptomascopus*, and most notably have a pair of distinct stridulatory files on abdominal tergite V similar in placement and structure to those of modern Nicrophorini, suggesting that their biology, including subsocial behavior, may have resembled that of modern Nicrophorini. Finally, mid-Cretaceous amber fossils from Myanmar (ca 100 Mya) resemble the modern genus *Nicrophorus* so closely, including having the unique lamellate antennal club of this genus, that they were referred to this genus (Cai et al. 2014). In contrast to all these fossils referable to Nicrophorini, fossils referable to Silphini, including those with long or entire elytra, are still not known until the mid–late Tertiary (ca 35 Mya or younger).

Based on this current state of knowledge of silphine fossils, we can conclude that the known age of silphines is comparable to that of the earliest known fossils reliably attributable to any other group of Staphylinidae, i.e., mid-Jurassic (e.g., Cai and Huang 2010, Chatzimanolis 2018, Cai et al. 2022). Older fossils of Triassic age (genus *Leehermania* Chatzimanolis et al.), originally attributed to Staphylinidae in Chatzimanolis (2018), were subsequently shown to belong to Hydroscaphidae (Fikáček et al. 2019). Furthermore, the earliest known silphines (i.e., all known Mesozoic fossils) have very truncate elytra and a robust habitus that at least superficially resembles many Jurassic Staphylinidae (e.g., Cai and Huang 2010) and even some modern Staphylinidae, including members of Apateticinae, Trigonurinae, some Omaliinae, and even large *Tachinus* spp. (Tachyporinae). These results are fully consistent with our conclusion from the review of modern phylogenetic analyses that Silphinae evolved from within Staphylinidae and share a suite of derived characters related to having truncate elytra as an ancestral feature. The longer elytra of many modern Silphini are thus likely to be a more recent and secondary development.

## ﻿Conclusion

From the multiple lines of phylogenetic evidence presented above, supported by the ever-expanding fossil record of Staphylinidae (29, possibly 30, of the 34 subfamilies now known), it seems well justified to treat Silphinae as a subfamily of a strongly supported monophyletic Staphylinidae.

## ﻿Additional information

### Author ORCIDs

Derek S. Sikes https://orcid.org/0000-0002-4336-2365

Margaret K. Thayer https://orcid.org/0000-0003-0061-9981

Alfred F. Newton https://orcid.org/0000-0001-9885-6306

### Data availability

All of the data that support the findings of this study are available in the main text and the respective data files of most of the analysis reviewed.

## References

[B1] Anderson RS (1982) Resource partitioning in the carrion beetle (Coleoptera: Silphidae) fauna of southern Ontario: ecological and evolutionary considerations. Canadian Journal of Zoology 60(6): 1314–1325. 10.1139/z82-178

[B2] Anderson RS Peck SB (1985) The Insects and Arachnids of Canada, Part 13. The carrion beetles of Canada and Alaska (Coleoptera: Silphidae and Agyrtidae). Publication 1778, Research Branch Agriculture Canada, Ottawa, 121 pp.

[B3] Avise JC (1986) Mitochondrial DNA and the evolutionary genetics of higher animals. Philosophical Transactions of the Royal Society of London. Series B, Biological Sciences 312(1154): 325–342. 10.1098/rstb.1986.0011 2870525

[B4] Ballard JWO Thayer MK Newton Jr AF Grismer ER (1998) Data sets, partitions, and characters: Philosophies and procedures for analyzing multiple data sets. Systematic Biology 47(3): 367–396. 10.1080/106351598260770 12066684

[B5] Beutel RG Leschen RA (2005) Phylogenetic analysis of Staphyliniformia (Coleoptera) based on characters of larvae and adults. Systematic Entomology 30(4): 510–548. 10.1111/j.1365-3113.2005.00293.x

[B6] Beutel RG Molenda R (1997) Comparative morphology of selected larvae of Staphylinoidea (Coleoptera, Polyphaga) with phylogenetic implications. Zoologischer Anzeiger 236: 37–67.

[B7] Bocak L Barton C Crampton‐Platt A Chesters D Ahrens D Vogler AP (2014) Building the Coleoptera tree‐of‐life for > 8000 species: Composition of public DNA data and fit with Linnaean classification. Systematic Entomology 39(1): 97–110. 10.1111/syen.12037

[B8] Buckley TR Simon C Flook PK Misof B (2000) Secondary structure and conserved motifs of the frequently sequenced domains IV and V of the insect mitochondrial large subunit rRNA gene. Insect Molecular Biology 9(6): 565–580. 10.1046/j.1365-2583.2000.00220.x 11122466

[B9] Cai C Huang D (2010) Current knowledge on Jurassic staphylinids of China (Insecta, Coleoptera). Earth Science Frontiers 17(Special Issue): 151–153.

[B10] Cai Y Li X (2021) The complete mitochondrial genome of a burying beetle, *Nicrophorusnepalensis* Hope, 1831 (Coleoptera: Silphidae). Mitochondrial DNA. Part B, Resources 6(6): 1727–1728. 10.1080/23802359.2021.1930220 PMC8158231 34104752

[B11] Cai C Thayer MK Engel MS Newton AF Ortega-Blanco J Wang B Wang X Huang D (2014) Early origin of parental care in Mesozoic carrion beetles. Proceedings of the National Academy of Sciences of the United States of America 111(39): 14170–14174. [online supporting information (9 pp.)] 10.1073/pnas.1412280111 25225362 PMC4191754

[B12] Cai C Tihelka E Giacomelli M Lawrence JF Ślipiński A Kundrata R Yamamoto S Thayer MK Newton AF Leschen RA Gimmel ML (2022) Integrated phylogenomics and fossil data illuminate the evolution of beetles. Royal Society Open Science 9(211771): 1–19. [Online supplement 87 pp.] 10.1098/rsos.211771 PMC8941382 35345430

[B13] Cameron SL (2014) Insect mitochondrial genomics: Implications for evolution and phylogeny. Annual Review of Entomology 59(1): 95–117. 10.1146/annurev-ento-011613-162007 24160435

[B14] Caterino MS Hunt T Vogler AP (2005) On the constitution and phylogeny of Staphyliniformia (Insecta: Coleoptera). Molecular Phylogenetics and Evolution 34(3): 655–672. 10.1016/j.ympev.2004.11.012 15683936

[B15] Chatzimanolis S (2018) A review of the fossil history of Staphylinoidea. In: Betz O Irmler U Klimaszewski J (Eds) Biology of rove beetles (Staphylinidae): Life history, evolution, ecology and distribution.Springer, Cham, Switzerland, 27–45. 10.1007/978-3-319-70257-5_3

[B16] Coiffait H (1972) Coléoptères Staphylinidae de la Région Paléarctique Occidentale. Généralités, sous-familles: Xantholininae et Leptotyphlinae. Nouvelle Revue d’Entomologie, Supplement 2(2): [ix+]651. https://www.persee.fr/doc/linly_0366-1326_1974_num_43_7_14125_t2_0047_0000_3

[B17] Crowson RA (1955) The natural classification of the families of Coleoptera. Nathaniel Lloyd and Co., London, 187 pp.

[B18] Crowson RA (1981) The biology of the Coleoptera. Academic Press, London, [xii +] 802 pp. 10.1016/C2013-0-07304-5

[B19] Dobler S Müller JK (2000) Resolving phylogeny at the family level by mitochondrial cytochrome oxidase sequences: Phylogeny of carrion beetles (Coleoptera, Silphidae). Molecular Phylogenetics and Evolution 15(3): 390–402. 10.1006/mpev.1999.0765 10860648

[B20] Erixon P Svennblad B Britton T Oxelman B (2003) Reliability of Bayesian posterior probabilities and bootstrap frequencies in phylogenetics. Systematic Biology 52(5): 665–673. 10.1080/10635150390235485 14530133

[B21] Feduccia A (2002) Birds are dinosaurs: Simple answer to a complex problem. The Auk 119(4): 1187–1201. https://www.jstor.org/stable/4090252

[B22] Fikáček M Beutel RG Cai C Lawrence JF Newton AF Solodovnikov A Ślipiński A Thayer MK Yamamoto S (2019 [2020]) Reliable placement of beetle fossils via phylogenetic analyses – Triassic *Leehermania* as a case study (Staphylinidae or Myxophaga?). Systematic Entomology 45(1): 175–187. [online supplements] 10.1111/syen.12386

[B23] Grebennikov VV Newton AF (2009) Good-bye Scydmaenidae, or why the ant-like stone beetles should become megadiverse Staphylinidae*sensu latissimo* (Coleoptera). European Journal of Entomology 106(2): 275–301. 10.14411/eje.2009.035

[B24] Grebennikov VV Newton AF (2012) Detecting the basal dichotomies in the monophylum of carrion and rove beetles (Insecta: Coleoptera: Silphidae and Staphylinidae) with emphasis on the Oxyteline group of subfamilies. Arthropod Systematics & Phylogeny 70(3): 133–165. 10.3897/asp.70.e31759

[B25] Gusarov VI (2018) Phylogeny of the family Staphylinidae based on molecular data: A review. In: Betz O Irmler U Klimaszewski J (Eds) Biology of Rove Beetles (Staphylinidae): Life History, Evolution, Ecology and Distribution.Springer, Cham, Switzerland, 7–25. 10.1007/978-3-319-70257-5_2

[B26] Hansen M (1997) Phylogeny and classification of the staphyliniform beetle families (Coleoptera). Biologiske Skrifter 48: 1–339. https://www.tandfonline.com/doi/pdf/10.1076/0165-0424(200006)22%3A3%3B1-I%3BFT242

[B27] Hatch MH (1927) Studies on the carrion beetles of Minnesota, including new species. Technical Bulletin, University of Minnesota Agricultural Experiment Station 48: 1–19. https://conservancy.umn.edu/bitstream/handle/11299/203988/mn1000_agexpstn_tb_048.pdf?sequence=1&isAllowed=y

[B28] Hoang DT Chernomor O von Haeseler A Minh BQ Vinh LS (2017) [2018]) UFBoot2: Improving the ultrafast bootstrap approximation. Molecular Biology and Evolution 35(2): 518–522. 10.1093/molbev/msx281 PMC5850222 29077904

[B29] Holloway AK Schnell GD (1997) Relationship between numbers of the endangered American burying beetle *Nicrophorusamericanus* Olivier (Coleoptera: Silphidae) and available food resources. Biological Conservation 81(1–2): 145–152. 10.1016/S0006-3207(96)00158-9

[B30] Hunt T Bergsten J Levkanicova Z Papadopoulou A St. John O Wild R Hammond PM Ahrens D Balke M Caterino MS Gómez-Zurita J Ribera I Barraclough TG Bocakova M Bocak L Vogler AP (2007) A comprehensive phylogeny of beetles reveals the evolutionary origins of a superradiation. Science 318(5858): 1913–1916. 10.1126/science.1146954 18096805

[B31] Ikeda H Nishikawa M Sota T (2012) Loss of flight promotes beetle diversification. Nature communications 3(1): 648. 10.1038/ncomms1659 [Corrigendum: 10.1038/ncomms1659 (23 October 2012)] PMC3272566 22337126

[B32] Inward D Beccaloni G Eggleton P (2007) Death of an order: A comprehensive molecular phylogenetic study confirms that termites are eusocial cockroaches. Biology Letters 3(3): 331–335. 10.1098/rsbl.2007.0102 17412673 PMC2464702

[B33] Irisarri I Meyer A (2016) The identification of the closest living relative (s) of tetrapods: Phylogenomic lessons for resolving short ancient internodes. Systematic Biology 65(6): 1057–1075. 10.1093/sysbio/syw057 27425642

[B34] Jakubec P Novák M Qubaiová J Šuláková H Růžička J (2019) Description of immature stages of *Thanatophilussinuatus* (Coleoptera: Silphidae). International Journal of Legal Medicine 133(5): 1549–1565. 10.1007/s00414-019-02040-1 30879134

[B35] Jeannel R Jarrige J (1949) Biospeologica LXVIII. Coléoptères Staphylinides (Première Série). Archives de Zoologie Expérimentale et Générale 86: 255–392.

[B36] Johnson KP Dietrich CH Friedrich F Beutel RG Wipfler B Peters RS Allen JM Petersen M Donath A Walden KK Kozlov AM Podsiadlowski L Mayer C Meusemann K Vasilikopoulos A Waterhouse RM Cameron SL Weirauch C Swanson DR Percy DM Hardy NB Terry I Liu S Zhou X Misof B Robertson HM Yoshizawa K (2018) Phylogenomics and the evolution of hemipteroid insects. Proceedings of the National Academy of Sciences of the United States of America 115(50): 12775–12780. 10.1073/pnas.1815820115 30478043 PMC6294958

[B37] Jordal BH Smith SM Cognato AI (2014) Classification of weevils as a data-driven science: Leaving opinion behind. ZooKeys 439: 1–18. 10.3897/zookeys.439.8391 PMC4196253 25317054

[B38] Kasule FK (1966) The subfamilies of the larvae of Staphylinidae (Coleoptera) with keys to the larvae of the British genera of Steninae and Proteininae. Transactions of the Royal Entomological Society of London 118(8): 261–283. 10.1111/j.1365-2311.1966.tb00838.x

[B39] Korte A Ribera I Beutel RG Bernhard D (2004) Interrelationships of Staphyliniform groups inferred from 18S and 28S rDNA sequences, with special emphasis on Hydrophiloidea (Coleoptera, Staphyliniformia). Journal of Zoological Systematics and Evolutionary Research 42(4): 281–288. 10.1111/j.1439-0469.2004.00282.x

[B40] Kypke JL (2018) Phylogenetics of the world’s largest beetle family (Coleoptera: Staphylinidae): A methodological exploration. PhD Thesis, University of Copenhagen, Copenhagen. vi, 106 pp. https://soeg.kb.dk/permalink/45KBDK_KGL/1pioq0f/alma99122777823905763

[B41] Lawrence JF Newton Jr AF (1982) Evolution and classification of beetles. Annual Review of Ecology and Systematics 13(1): 261–290. 10.1146/annurev.es.13.110182.001401

[B42] Lawrence JF Ślipiński A Seago AE Thayer MK Newton AF Marvaldi AE (2011) Phylogeny of the Coleoptera based on morphological characters of adults and larvae. Annales Zoologici 61(1): 1–217. 10.3161/000345411X576725

[B43] Liu Y Tihelka E Thayer MK Newton AF Huang D Tian L Cai C (2021) A transitional fossil sheds light on the early evolution of the Staphylinine group of rove beetles (Coleoptera: Staphylinidae). Journal of Systematic Palaeontology 19(4): 321–332. 10.1080/14772019.2021.1917705

[B44] Lü L Cai CY Zhang X Newton AF Thayer MK Zhou HZ (2019) [2020] Linking evolutionary mode to palaeoclimate change reveals rapid radiations of staphylinoid beetles in low-energy conditions. Current Zoology 66(4): 435–444. 10.1093/cz/zoz053 32617092 PMC7319441

[B45] Maddison WP Maddison DR (2018) Mesquite: a modular system for evolutionary analysis. Version 3.6. http://www.mesquiteproject.org

[B46] McKenna DD Farrell BD Caterino MS Farnum CW Hawks DC Maddison DR Seago AE Short AEZ Newton AF Thayer MK (2015) Phylogeny and evolution of Staphyliniformia and Scarabaeiformia: Forest litter as a stepping stone for diversification of nonphytophagous beetles. Systematic Entomology 40(1): 35–60. 10.1111/syen.12093

[B47] McKenna DD Shin S Ahrens D Balke M Beza-Beza C Clarke DJ Donath A Escalona HE Friedrich F Letsch H Liu S Maddison D Mayer C Misof B Murin PJ Niehuis O Peters RS Podsiadlowski L Pohl H Scully ED Yan EV Zhou X Ślipiński A Beutel RG (2019) The evolution and genomic basis of beetle diversity. Proceedings of the National Academy of Sciences of the United States of America 116(49): 24729–24737. 10.1073/pnas.1909655116 31740605 PMC6900523

[B48] Naomi SI (1985) The phylogeny and higher classification of the Staphylinidae and their allied groups (Coleoptera, Staphylinoidea). Esakia 23: 1–27. 10.5109/2464

[B49] Newton AF (2022) StaphBase: Staphyliniformia world catalog database (version Aug 2022). In: Bánki O, Roskov Y, et al. (Eds) Catalogue of Life Checklist (Aug 2022). https://www.catalogueoflife.org/ [(accessed 23 February 2024) 10.48580/dfqf-3gk]

[B50] Peck SB (2000) [2001] Silphidae Latreille, 1807. In: Arnett RH, Thomas MC (Eds) American Beetles: Archostemata, Myxophaga, Adephaga, Polyphaga: Staphyliniformia. Vol.1. CRC Press, Boca Raton, Florida, USA 268–271.

[B51] Peck SB Anderson RS (1985) Taxonomy, phylogeny and biogeography of the carrion beetles of Latin America (Coleoptera: Silphidae). Quaestiones Entomologicae 21(3): 247–317.

[B52] Sikes DS (2016) Silphidae Latreille, 1807. In: Beutel RG Leschen RAB (Eds) Coleoptera, beetles volume I: Morphology and systematics (Archostemata, Adephaga, Myxophaga, Polyphaga partim) 2nd edn.Handbook of Zoology, Arthropoda: Insecta (Beutel RG, Kristensen NP, eds). Walter de Gruyter, Berlin, Germany, 386–394.

[B53] Sikes DS Venables C (2013) Molecular phylogeny of the burying beetles (Coleoptera: Silphidae: Nicrophorinae). Molecular Phylogenetics and Evolution 69(3): 552–565. 10.1016/j.ympev.2013.07.022 23911726

[B54] Sikes DS Madge RB Newton AF (2002) A catalog of the Nicrophorinae (Coleoptera: Silphidae) of the world. Zootaxa 65(1): 1–304. 10.11646/zootaxa.65.1.1

[B55] Sohn JC Nam GS (2021) New fossil genus and species of carrion beetle (Coleoptera, Silphidae) from the Lower Cretaceous Jinju Formation, South Korea. Journal of Asia-Pacific Entomology 24(3): 584–587. 10.1016/j.aspen.2021.05.003

[B56] Song N Zhai Q Zhang Y (2021) Higher-level phylogenetic relationships of rove beetles (Coleoptera, Staphylinidae) inferred from mitochondrial genome sequences. Mitochondrial DNA. Part A, DNA Mapping, Sequencing, and Analysis 32(3): 98–105. 10.1080/24701394.2021.1882444 33570440

[B57] Timmermans MJTN Barton C Haran J Ahrens D Culverwell CL Ollikainen A Dodsworth S Foster PG Bocak L Vogler AP (2016) Family-level sampling of mitochondrial genomes in Coleoptera: Compositional heterogeneity and phylogenetics. Genome Biology and Evolution 8(1): 161–175. 10.1093/gbe/evv241 PMC4758238 26645679

[B58] Toussaint EFA Seidel M Arriaga-Varela E Hájek J Král D Sekerka L Short AEZ Fikáček M (2016) The peril of dating beetles. Systematic Entomology 42(1): 1–10. 10.1111/syen.12198

[B59] Trumbo ST Sikes DS Philbrick PKB (2016) Parental care and competition with microbes in carrion beetles: A study of ecological adaptation. Animal Behaviour 118: 47–54. 10.1016/j.anbehav.2016.06.001

[B60] Vences M Guayasamin JC Miralles A De La Riva A (2013) To name or not to name: Criteria to promote economy of change in Linnaean classification schemes. Zootaxa 3636(2): 201–244. 10.11646/zootaxa.3636.2.1 26042291

[B61] von Reumont BM Jenner RA Wills MA Dell’Ampio E Pass G Ebersberger I Meyer B Koenemann S Iliffe TM Stamatakis A Niehuis O Meusemann K Misof B (2012) Pancrustacean phylogeny in the light of new phylogenomic data: Support for Remipedia as the possible sister group of Hexapoda. Molecular Biology and Evolution 29(3): 1031–1045. 10.1093/molbev/msr270 22049065

[B62] Yamamoto S (2021) Tachyporinae revisited: Phylogeny, evolution, and higher classification based on morphology, with recognition of a new rove beetle subfamily (Coleoptera: Staphylinidae). Biology 10(323): 1–156. [online supplements] 10.3390/biology10040323 PMC8069000 33924435

[B63] Zhang X Zhou HZ (2013) How old are the rove beetles (Insecta: Coleoptera: Staphylinidae) and their lineages? Seeking an answer with DNA. Zoological Science 30(6): 490–501. 10.2108/zsj.30.490 23721473

[B64] Zhang S-Q Che L-H Li Y Liang D Pang H Ślipiński A Zhang P (2018) Evolutionary history of Coleoptera revealed by extensive sampling of genes and species. Nature Communications 9(205): 205. 10.1038/s41467-017-02644-4 PMC5768713 29335414

[B65] Zhao TY He L Xu X Chen ZN Gao YY Liang L (2022) The first mitochondrial genome of *Creophilus* Leach and *Platydracus* Thomson (Coleoptera: Staphylinidae: Staphylinini) and phylogenetic implications. Zootaxa 5099(2): 179–200. 10.11646/zootaxa.5099.2.2 35391419

